# Machine learning in precision diabetes care and cardiovascular risk prediction

**DOI:** 10.1186/s12933-023-01985-3

**Published:** 2023-09-25

**Authors:** Evangelos K. Oikonomou, Rohan Khera

**Affiliations:** 1 Section of Cardiovascular Medicine, Department of Internal Medicine, Yale School of Medicine, New Haven, CT USA; 2 Section of Health Informatics, Department of Biostatistics, Yale School of Public Health, New Haven, CT USA; 3 Section of Biomedical Informatics and Data Science, Yale School of Medicine, New Haven, CT USA; 4 Center for Outcomes Research and Evaluation, Yale-New Haven Hospital, 195 Church St, 6th floor, New Haven, CT 06510 USA

**Keywords:** Machine learning, Artificial intelligence, Prediction, Personalized medicine, Digital health, Diabetes, Cardiovascular disease

## Abstract

Artificial intelligence and machine learning are driving a paradigm shift in medicine, promising data-driven, personalized solutions for managing diabetes and the excess cardiovascular risk it poses. In this comprehensive review of machine learning applications in the care of patients with diabetes at increased cardiovascular risk, we offer a broad overview of various data-driven methods and how they may be leveraged in developing predictive models for personalized care. We review existing as well as expected artificial intelligence solutions in the context of diagnosis, prognostication, phenotyping, and treatment of diabetes and its cardiovascular complications. In addition to discussing the key properties of such models that enable their successful application in complex risk prediction, we define challenges that arise from their misuse and the role of methodological standards in overcoming these limitations. We also identify key issues in equity and bias mitigation in healthcare and discuss how the current regulatory framework should ensure the efficacy and safety of medical artificial intelligence products in transforming cardiovascular care and outcomes in diabetes.

**Supplementary Information:**

The online version contains supplementary material available at 10.1186/s12933-023-01985-3.

## Introduction

The rapid progress in artificial intelligence (AI) and machine learning (ML) has raised hopes for a more personalized, efficient, and effective approach to the management of diabetes mellitus and its cardiovascular sequelae [1, 2]. It is estimated that nearly 529 million people worldwide and 35 million Americans currently have diabetes, with cardiovascular disease (CVD) representing the leading cause of morbidity and mortality [3, 4]. Recognizing the need for improvement in the diagnosis, monitoring, and treatment of this growing patient population, AI and ML have already been applied to automate the screening of diabetes, detect macrovascular and microvascular complications [5–11], and enable multiomic phenotyping for personalized prevention and therapy recommendations [12, 13].

Unfortunately, most AI and ML-based tools fail to translate into improved outcomes for our patients and communities. This gap between evidence generation and clinical implementation is exemplified by the subpar real-world uptake of multiple therapies that reduce cardiovascular risk [14–17]. Furthermore, the current paradigm of medical AI heavily relies on existing data streams that reflect and thus perpetuate systemic biases. Acknowledging these limitations is necessary to prevent the misuse and overuse of AI and ML in medicine and further underscores the need for good research practices to ensure reproducibility [18] as well as guide the practical, ethical [19], and regulatory challenges that arise from the burgeoning use of these technologies [20, 21].

In this comprehensive review, we offer a broad overview of the various ML methods and how they may be leveraged in developing predictive models. This review focuses on these methods in the context of ML in the diagnosis, prognostication, phenotyping, and treatment of diabetes and its cardiovascular complications. In addition to discussing the properties of the models that enable their successful application in complex risk prediction, we define challenges that arise from the misuse of AI and ML, and the role of methodological standards in combating these challenges. We also identify key issues on equity and bias mitigation in healthcare and ways in which the regulatory framework can ensure the efficacy and safety of ML and AI in transforming cardiovascular care and outcomes in diabetes.

## Developing and evaluating clinical machine learning models

An understanding of the principal tenets of model development and evaluation is essential for interpreting the evidence. These concepts are broad, applicable across a range of clinical conditions and ML tasks and represent the foundations of critical AI and ML appraisal.

### Artificial intelligence (AI) and machine learning (ML)

Though AI and ML are inextricably linked, they are not identical terms [22, 23]. *Artificial intelligence* (AI) describes the ability of a machine to perform tasks that are typical of human intelligence, such as understanding natural language, problem-solving, or creative tasks like generating images and text. On the other hand, the process through which an AI system acquires this ability, learning and improving from experience and observed data to make predictions about new or unseen cases, is called *machine learning* (ML).

### Model training

The specific step during which a model learns from data is also known as *training*, whereas the respective dataset is referred to as *training set*. Here, the model makes predictions and subsequently adjusts its parameters based on a metric that quantifies how good or bad the predictions are (*loss function*). It is typical that during training, the model will be applied to an unseen group of observations (*validation set*) to get a more reliable assessment on performance on unseen data. Further testing in external sets drawn from geographically and temporally distinct populations can serve to solidify claims about external model validity [24].

### Supervised and unsupervised learning

The learning process can be supervised or unsupervised [22]. *Supervised learning* describes an iterative process that selects relevant input features and then assigns weights to link the input data to a given value (regression) or class (classification). *Unsupervised learning,* on the other hand, analyzes and clusters unlabeled datasets by identifying similarities and dissimilarities between data points, therefore uncovering hidden patterns in the data. These two approaches should be considered complementary and are often used in conjunction to address distinct problems. Supervised learning can be used to better predict future cardiovascular risk (regression), or the presence of diabetic retinopathy (classification), whereas unsupervised approaches can be used to identify distinct phenotypic clusters of patients with diabetes with differences in baseline risk, prognosis, and treatment response.

Building on these concepts, *self-supervised learning* (SSL) processes unlabeled data to create key representations that can facilitate downstream tasks [25]. In principle, SSL closely resembles unsupervised learning since it is applied to unlabeled data. However, instead of focusing on tasks like clustering, SSL attempts to solve tasks traditionally addressed through supervised learning, such as classification and regression [26]. We [27, 28], and others [29, 30], have shown that this is a powerful method to train clinical models, especially when there is a paucity of high-quality labels.

### Machine learning algorithms

**Fig. 1:**
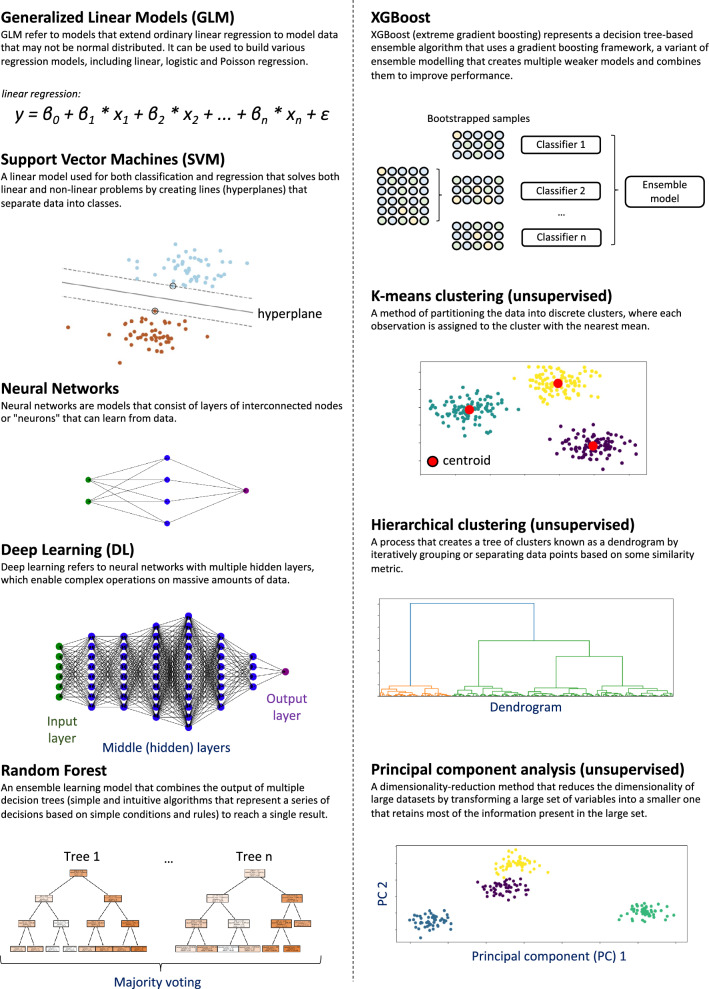
Overview of commonly used algorithms in medical machine learning

Whether supervised or unsupervised, the ML process requires a set of rules and statistical techniques that can learn meaningful patterns from data, known as *algorithms*. A representative list of ML algorithms used in medical applications is shown in Fig. 1. Ranging from linear regression to deep learning algorithms, these vary substantially in their ability to model complex data, interpretability, and performance [22]. Further, they can be adapted to model not only cross-sectional or short-term outcomes, as done with logistic regression, but also long-term predictions through survival analysis, similar to Cox regression modeling which has been widely used to estimate CVD risk in the US [31], UK [32], and Europe [33]. Some notable examples include (survival) random forests and deep learning algorithms, which have all been adapted to model long-term hazards [34–36].

### Assessing model performance

**Fig. 2:**
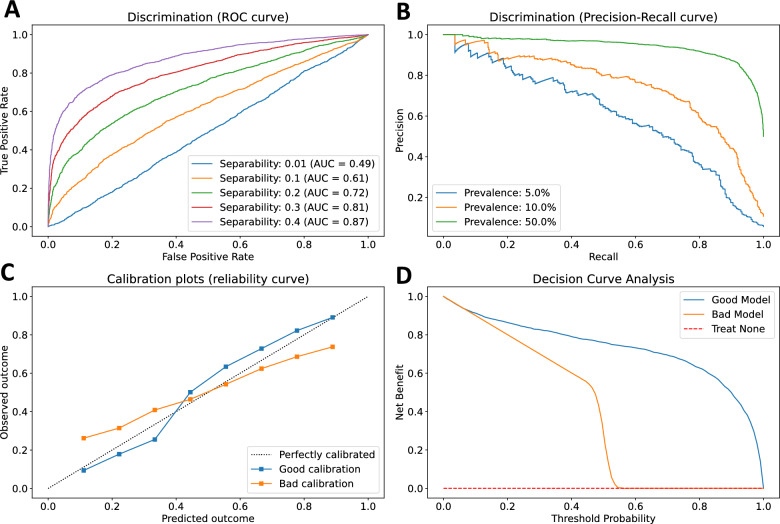
Discrimination, calibration, and net clinical benefit. The comprehensive evaluation of a predictive model requires the simultaneous evaluation of its discrimination, calibration, and incremental value beyond the current standard-of-care. **A** The area under the receiver operating characteristic curve (AUROC) reflects the trade-off between sensitivity (true positive rate) and specificity (1-false positive rate) at different thresholds and provides a measure of separability, in other words the ability of the model to distinguish between classes (0.5 = no separation, 1 = perfect separation). **B** Models with similar AUROC may exhibit different behavior when the prevalence of the label varies. The precision–recall curve demonstrates the trade-off between the positive predictive value (*precision*) and sensitivity (*recall*), and illustrates how the area under the curve may vary substantially as the prevalence of the label of interest decreases from 50 to 5%. **C** Models with similar AUROC may also differ in their calibration. A model with good calibration (i.e. blue line) makes probabilistic predictions that match real world probabilities. On the other hand, the model shown in orange underestimates and overestimates risk at lower and higher prediction thresholds, respectively. **D** Finally, models should be compared against established standard-of-cares while incorporating clinical consequences and comparing the net clinical benefit across varying risk levels to established or no risk stratification approaches. Curves were generated using synthetic datasets for illustration purposes

The comprehensive evaluation of the performance of a predictive ML model requires an integrated assessment of discrimination, calibration, and clinical benefit (Fig. 2; a detailed table of metrics used in classification and regression tasks, along with their strengths and weaknesses is also presented in Additional file 1: Table S1) [37]. Commonly used *discrimination* metrics, such as the Area Under the Receiver Operating Characteristic curve (AUROC) and the Area Under the Precision–Recall Curve (AUPRC) describe the model’s ranking of individual predictions and ability to discriminate between different classes. However, AUROC may not offer a complete description of the model’s performance, particularly in imbalanced datasets. For instance, a model with 95% sensitivity and specificity screening for a rare label (e.g., screening for type 1 diabetes mellitus in the community, prevalence ~ 0.55%), a positive prediction is more likely to be false positive than true positive. *Calibration* assesses the agreement between the predicted and observed outcomes across the entire range of predictions [38]. Two models may have a similar AUROC but may differ substantially in their calibration performance, a crucial difference that may often impact clinical decision-making if predictions consistently overestimate or underestimate risk. Guidelines on good research practices in prediction modeling suggest that for any given model both discrimination and calibration are reported [39]. Moreover, a complex model may have good discriminatory performance but lack incremental value beyond a simpler or established model, something that can be further evaluated by metrics such as the Net Reclassification Improvement (NRI) and Integrated Discrimination Improvement (IDI) [37]. Finally, good discrimination and calibration do not necessarily translate into net clinical benefit for a specific clinical application. In this setting, decision curve analysis (DCA) can be used to assess the net benefit of a model across a range of potential thresholds for action, weighing the benefit versus harm versus alternative risk stratification approaches [40].

### Interpretability and explainability

Human end-users may feel reluctant to use what they cannot understand. *Interpretability* and *explainability* describe two closely linked yet slightly different concepts [41, 42]. *Interpretability* refers to the extent to which a human can understand how features are combined to make predictions. This is particularly desirable when training a model to understand its general behavior and identify potential sources of bias. *Explainability* is a property of ML models that describes the extent to which the inner workings of a model can be explained in human terms, that is, understanding why a model made a specific prediction (often on a case-by-case basis). This is important for the end-user and has practical and regulatory implications. A model can be interpretable without being explainable, and vice versa; however, ideally, a model should be both [43].

## Data-driven advances in diabetes and cardiovascular disease

From continuous glucose monitoring devices to electronic health records (EHR), electrocardiograms (ECG), retinal images, and computed tomography images, the day-to-day monitoring, screening, and management of patients with diabetes have a constant stream of structured and unstructured data. The following section illustrates the breadth of tools that are being developed for use by individuals and their healthcare teams.

### Targeted screening and risk stratification of prediabetes and diabetes

Both the American Diabetes Association (ADA) [44], and the United States Preventive Services Task Force (USPTF) [45] have emphasized the importance of early screening for pre-diabetes and diabetes among asymptomatic adults to ensure timely diagnosis and prevent downstream diabetes complications and its sequalae. Current guidelines recommend screening for pre-diabetes and type 2 diabetes with an informal assessment of risk factors or a validated risk calculator among all asymptomatic adults and testing among adults of any age who are overweight or obese and have one or more risk factors [44]. Several risk scores have been proposed for a more personalized risk assessment of type 2 diabetes, such as the American Diabetes Association questionnaire, a logistic regression model trained in National Health and Nutrition Examination Survey (NHANES), Atherosclerosis Risk in Communities (ARIC) and Cardiovascular Health Study (CHS) studies with a reported AUROC of 0.79 to 0.82 for undiagnosed diabetes among U.S. adults aged 20 years or older [46], the Australian type 2 diabetes risk Assessment Tool (AUSDRISK) to predict the incidence of type 2 diabetes over 5 years among participants 25 years or older (AUROC of 0.78) [47], and the Cambridge Risk score to detect cross-sectionally elevated HbA1c levels among individuals aged 45 years (AUROC of 0.84 for HbA1c 7% or greater) [48]. The moderate accuracy of these tools in addition to concerns about their external validity [49] has prompted researchers to explore whether targeted screening could be improved through ML of structured and unstructured data [5–11].

In NHANES, an XGBoost classifier based on 123 variables showed an AUROC of 0.86 in detecting the presence of an established diabetes diagnosis, though the performance dropped to 0.73 when detecting undiagnosed diabetes adjudicated based on abnormal laboratory findings [50]. In a retrospective analysis of 16 predictors from routine health check-up data of 277,651 participants from Japan, a light gradient boosting machine algorithm was able to predict the 3-year incidence of diabetes with an AUROC of 0.84, demonstrating significantly improved performance compared with a logistic regression model for large training populations of 10,000 patients or more [6]. Another ML algorithm built using EHR and administrative healthcare data from Canada reportedly identified type 1 diabetes cases with 87.2% sensitivity and 99.9% specificity [51].

Moving beyond EHR models relying on administrative datasets, an analysis of 1262 individuals from India showed that an XGBoost algorithm using ECG inputs had excellent performance (97.1% precision, 96.2% recall) and good calibration in detecting type 2 diabetes and pre-diabetes [7]. Furthermore, the integration of a genome-wide polygenic risk score and serum metabolite data with structured clinical parameters using a random forest model resulted in improved type 2 diabetes risk prediction in a Korean cohort of 1425 participants [8]. Several other studies have also defined metabolomic [52] and proteomic signatures for identifying diabetes or insulin resistance [9]. Integrative personal omics profiles (iPOP) that combine genomic, transcriptomic, proteomic, metabolomic, and autoantibody profiles from a single individual over several months can also be harnessed to connect genomic information with dynamic omics activity, describe host-microbiome interactions, and describe personal aging markers, thus risk stratifying various medical risks, including type 2 diabetes [53–57].

Finally, imaging-based biomarkers are also being studied, with the development of DL models that can automatically segment and output measurements of pancreatic attenuation, volume, fat content, fractal dimension, and parameters associated with visceral adiposity and muscle attenuation/volume. For instance, in a retrospective cohort of 8992 patients undergoing screening CT colonography, a DL model of the above phenotypes could predict the future incidence of diabetes with an AUROC of 0.81 to 0.85 [10].

### Computable phenotypes of patients with diabetes

The traditional classification of diabetes into type 1 and type 2 does not fully capture the complex and highly heterogeneous nature of the condition. From the heterogeneity of the islet microenvironment to the diversity of pathophysiological endotypes that span multiple demographic groups, diabetes mellitus affects a diverse group of patients with distinct molecular underpinnings that require individualized approaches to therapy [58]. Multiomic signatures may provide insights into the interaction of a patient’s genome, phenome, and environment [53–57], but are often hard to measure at scale.

Unsupervised ML techniques using routinely available EHR computable phenotypes can provide valuable data-driven inference about distinct phenotypic clusters. In longitudinal DL-based clustering of 11,028 patients with type 2 diabetes using a kernelized autoencoder algorithm that mapped 5 years of data, there were seven phenotypic clusters with distinct clinical trajectories and varying prevalence of comorbidities (i.e., hypertension, hypercholesterolemia) or diabetic complications [13]. In a separate analysis of 8,980 patients with newly diagnosed diabetes from Sweden, k-means and hierarchical clustering revealed five replicable clusters with significant differences in the observed risk of diabetic complications [59]. A separate analysis of 175,383 patients with type 2 diabetes further identified 20 frequent comorbidity clusters, and using Bayesian nonparametric models demonstrated a complex and dynamic interrelationship between diabetes-related comorbidities and accelerated disease progression [60].

Despite this, computable definitions can vary significantly in their ability to capture the respective phenotypes. Such differences can have a substantial impact on model performance even within the same center. In an analysis of 173,503 adults from the Duke University Health System, the concordance of variable definitions of diabetes (with or without ICD-9-CM codes) ranged from 86% to as low as 50% [61].

Finally, DL approaches, such as natural language processing, can be used to screen large hospital registries to monitor the quality of care. As shown in an analysis of 33,461 patients with diabetes from 2014 to 2020 in Northern California, a natural language processing approach accurately identified statin nonuse (AUROC of 0.99 [0.98–1.00]) and highlighted patient- (side effects/contraindications), clinician- (guideline‐discordant practice), and system-centered reasons (clinical inertia) for statin nonuse, with notable variation by race and ethnicity [62].

### Predicting CVD among patients with diabetes (from diagnosis to risk prediction)

Diabetes is associated with a range of micro- and macro-vascular complications [3]. Given their simplicity and wide availability, fundoscopic images were used in some of the earliest DL models in medicine, predicting diabetic retinopathy with performance matching that of expert readers [11, 63, 64]. This has opened the way for efficient screening of both diabetes, and diabetes-related chronic kidney disease and retinopathy in settings with limited resources, as demonstrated in real-world implementation studies in Thailand and India [65, 66].

While current guidelines endorse routine screening for microvascular complications, there is a paucity of data to support the routine screening for macrovascular complications in asymptomatic individuals [67]. Diabetes has traditionally been regarded as an atherosclerotic CVD (ASCVD) equivalent [44]. Non-invasive cardiovascular imaging approaches, such as measurement of coronary artery calcium (CAC) [68], coronary computed tomography angiography [69], or functional testing [67], are often used to further risk stratify patients with diabetes and diagnosed subclinical CVD. However, such approaches are costly and hard to implement for population-level screening.

ML approaches may support more efficient screening of CVD in this population. In an analysis of NHANES, logistic regression, SVM, XGBoost and random forest models, as well as an ensemble of the four, showed comparable performance in detecting CVD among all-comers with an AUROC of 0.81 to 0.83 [50]. In a separate single-center study from China, training a model to predict the co-occurrence of coronary heart disease and diabetes using 52 structured features in 1273 patients with type 2 diabetes resulted in an AUROC of 0.77–0.80; however, this dropped to 0.7 in an independent dataset, highlighting the challenges in the generalizability of such tools when trained in single-center cohorts [70]. In a retrospective analysis of administrative data from Australia, investigators combined ML techniques with a social network analytic approach to define the disease network for patients with diabetes with or without CVD and identify discriminatory features for CVD presence, with a reported overall AUROC of 0.83 for the random forest classifier, only dropping to 0.81 for a logistic regression model [71]. Overall, it appears that in most cohorts there were minimal gains from using more complex and less interpretable algorithms compared to standard logistic regression. Moreover, many studies do not report the incremental value beyond established risk scores such as the pooled cohort equations, which prevents a reliable assessment of their net clinical value.

Predictive models can further be customized for more specific cardiovascular conditions, such as congestive heart failure, and model survival analyses incorporating time-to-event outcomes. In a post hoc analysis of the ACCORD trial, a non-parametric random survival forest model predicted the risk of incident heart failure, with a C-statistic of 0.77 [72]. A parsimonious five-feature integer-based score based on this model maintained moderate discriminatory performance in both ACCORD (Action to Control Cardiovascular Risk in Diabetes) and an independent test set from Antihypertensive and Lipid-Lowering Treatment to Prevent Heart Attack Trial (ALLHAT) with an AUROC of 0.74 and 0.70, respectively. In a separate retrospective analysis of an EHR-based cohort of patients with diabetes undergoing cardiac testing, a deep neural network survival method resulted in an AUROC of 0.77 for incident heart failure [36].

Many of these studies should be interpreted with caution. First, as shown in a recent systematic review of predictive models for the detection of CVD among patients with diabetes, there is often a high risk of bias in several studies and poor adherence to standardized reporting guidelines [73–75]. Second, retrospective analyses of single-center cohorts and administrative claims are also prone to perpetuating biases and inequities in healthcare since patients who have better access to healthcare are more likely to utilize such resources and be represented in administrative claims datasets [76].

### Digital health for diabetes care optimization and personalization through predictive algorithms

As the focus shifts from the secondary prevention of diabetes-related complications to earlier prevention in the community, various digital health technologies emerge that can be deployed at scale and minimal cost. Large language models (LLM) have already led to smart conversational agents (“chatbots”), such as ChatGPT, which are freely accessible to most individuals with internet access. Such models are task-agnostic and have been shown to provide “concise”, “well-organized” and easy-to-understand instructions to a series of questions regarding diabetes self-management, albeit with occasional factual inaccuracies [77]. Such tools could be incorporated into existing digital healthcare platforms that combine glucometer, bioelectrical impedance, blood pressure, activity, and AI-derived nutritional analysis data to improve glycemia and weight loss among patients with type 2 diabetes [78].

In the same vein, the rapid uptake in the use of wearable devices and smartwatches has led to AI-enabled solutions to optimize glycemic management. These applications have built on an expanding body of research highlighting the value of AI-ECG (both using 12-lead or 1-lead signals) in detecting subclinical forms of cardiomyopathy and arrhythmias [79–81]. Recent work from our lab has further expanded these approaches to the use of ECG images [82, 83] and single-lead wearable signals [84], enabling the scaling of such technologies to low-resource settings and to ubiquitous data streams.

In diabetes, AI-ECG-guided monitoring through customized DL models has shown promise in detecting hypoglycemic events [85, 86], and is currently being studied in prospective studies [87, 88]. In one of the pilot studies, investigators trained personalized models using a combination of convolutional (CNN) and recurrent neural networks (RNN) for each participant using data collected over the run-in period, followed by subsequent testing in the same patient. This combination of distinct model architectures takes advantage of the distinct strengths of each model type, with CNN learning hierarchical, abstract representations of the input space, whereas RNN learns sequence patterns across time [85]. Similar concepts have been applied to continuous glucose monitoring (CGM). Here, multi-modal data integration from CGM devices, meal or insulin entries and sensor wristbands has shown promise in detecting hypo- or hyperglycemic events in patients with type 1 diabetes in both simulation [89], as well as small real-world prospective studies [90]. While such technologies have the potential to democratize access to high-value care, we have shown that as of 2020 use patterns suggested disproportionately lower use among individuals with or at risk of CVD than those without CVD risk factors, with fewer than 1 in 4 using such devices [91, 92].

## AI-driven innovation in clinical trials and evidence generation

### Detecting heterogeneous treatment effects

Randomized controlled trials (RCTs) represent the methodological and regulatory gold-standard to test the efficacy and safety of new therapies [93]. However, RCTs traditionally report an average treatment effect (ATE) which does not adequately describe the individualized benefit for each unique patient profiles [94]. The detection of reliable heterogeneous treatment effects (HTE) is limited by the fact that in outcomes trials participants get assigned to one arm (thus the “counterfactual” is never observed). In addition, most trials lack statistical power to detect subgroup differences [95].

One way to explore such differences is through a priori or post hoc-defined clinical subgroups. For instance, it has been shown that sex and body mass index are associated with heterogeneity in the treatment and safety signal of thiazolidinediones and sulfonylureas [96], whereas insulin resistance has been associated with significant differences in the glycemic response to dipeptidyl peptidase 4 (DPP-4) inhibitors [97]. Such subgroups, however, rely on simplistic subgroup definitions, and may not accurately reflect the phenotypic diversity seen in clinical profiles and treatment response. Various statistical approaches have been employed to identify such complex effects, with methods ranging from unsupervised clustering [98] to causal forests and meta-learners (i.e. X-learners), algorithms that can use any supervised learning or regression method to estimate the *conditional* average treatment effect [94]. In applying this approach in ACCORD and Veteran Affairs Diabetes Trial (VADT), investigators described eight subgroups in which the differences in major adverse cardiovascular events ranged from as low as -5.1% to as high as 3.1% [99].

**Fig. 3:**
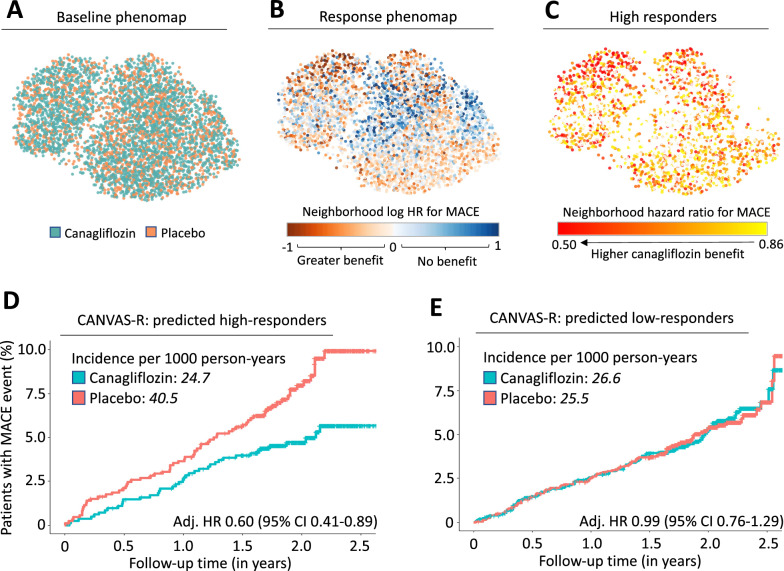
Phenomapping-derived tools for personalized effect estimates. Phenomaps enable a visual and topological representation of the baseline phenotypic variance of a trial population while accounting for many pre-randomization features. As shown in an analysis of the Canagliflozin Cardiovascular Assessment (CANVAS) trial [138], a phenomap representation of all enrolled patients shows that the study arms are randomly distributed in the phenotypic space (**A**). Through a series of iterative analyses centered around each patient’s unique phenotypic location, a machine learning model can learn phenotypic signatures associated with distinct responses to canagliflozin versus placebo therapy (**B**, **C**). An extreme gradient boosting algorithm trained to describe this heterogeneity in treatment effect in CANVAS successfully stratified the independent CANVAS-R population into high- (**D**) and low-responders (**E**). Panels reproduced with permission from Oikonomou et al. [12]

Most of these approaches focus on the absolute risk reduction of a therapy, which reflects both the relative treatment effect as well as a patient’s baseline risk. We previously developed and tested a phenomapping-based method that creates multidimensional representations of a population based on the full breadth of pre-randomization phenotypes. Over a series of in silico simulations that account for the unique phenotype of each participant relative to all other participants, an ML algorithm learns signatures that are consistently associated with a higher or lower relative treatment effect. In representative applications, our approach has reproduced a beneficial association between the use of anatomical as opposed to functional testing in patients with diabetes and chest pain [100, 101], and highlighted heterogeneity across phenotypes in the cardiovascular benefits of canagliflozin [12] as well as intensive systolic blood pressure control [102] (Fig. 3). However, prospective validation of any post-hoc comparisons is required to inform treatment decisions.

### Towards smarter clinical trials

**Fig. 4:**
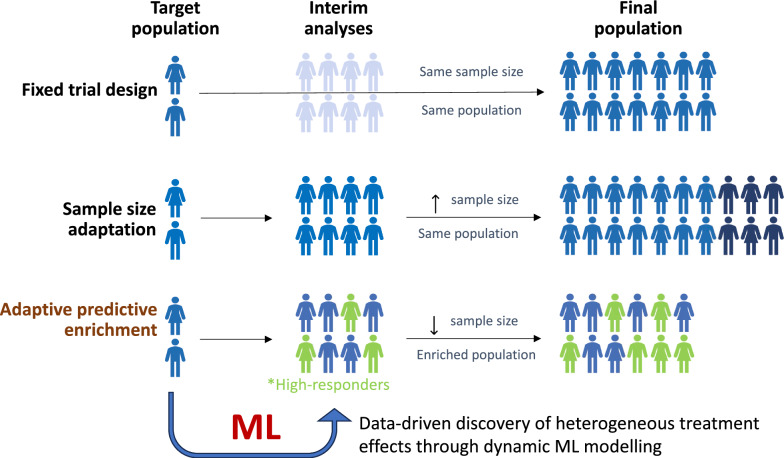
Machine learning for predictive enrichment of randomized control trials. Machine learning can be used to guide adaptive clinical trial design though data-driven inference and predictive enrichment. Traditional fixed trial designs do not allow modifications in the patient population, whereas sample size adaptations only allow interim revisions in the power calculations and target sample sizes based on the accumulating rate of primary outcome and safety events. In trials whereas there happens to be clinically meaningful heterogeneity in the treatment effect, a priori inclusion of machine learning, data-driven inference may provide early signals of heterogeneous benefit or harm and a reference for adaptive predictive enrichment. This approach can optimize the trial’s efficacy, shorten its duration, minimize its costs, maximize inference, and ultimately ensure safety for the study participants. *ML* machine learning

Furthermore, ML can be used to guide the design of adaptive clinical trials, guiding protocol modifications based on accumulating data [103] (Fig. 4). The need for methodological innovation in this space has been embraced by the United States Food and Drug Administration (FDA) [104]. For instance, ML-driven approaches of individualized predictive benefit could be integrated into interim analyses to prioritize randomization of patients with a higher expected net clinical benefit from the studied intervention [105]. In a simulation of real-world clinical trial data from Insulin Resistance in Stroke study (IRIS) [106], and Systolic Blood Pressure Intervention Trial (SPRINT) [107] an ML-informed strategy of adaptive, predictive enrichment enabled a consistent reduction in the number of enrolled participants, while preserving the original trial’s effect [105].

### Causal inference from observational data

Modern RCTs are both resource and time-intensive [108], particularly when evaluating the effects of novel therapies on major clinical endpoints [109–111]. Federated analytic approaches that utilize large-scale, multinational, real-world databases, such as the Large-scale evidence generation and evaluation across a network of databases for type 2 diabetes mellitus (LEGEND-T2DM) initiative, are currently underway to enable comparative effectiveness analysis through both traditional and ML-driven big data approaches [112, 113].

## Key methodological considerations when interpreting ML models

### Finding the best algorithm

In the ML literature, it is widely recognized that a priori knowledge of an optimal algorithm is challenging for any given task. This often depends on the underlying dataset, the performance metric utilized, whereas for any given algorithm performance may still vary with tuning of model-specific hyperparameters (values used to control the training process that are external to the model and cannot be computed from the data). Second, there is a common misconception that for any given dataset, complex models will always outperform less complex ones. This may be true for tasks involving unstructured data, in particular biomedical images and videos, where DL models enable automatic learning and hierarchical combination of key spatial as well as temporal features (rather than relying on hand-engineered features), as well as models that are robust to variations, scalable and transferrable across tasks [114]. However, for structured datasets, such as databases of clinical information used for predictive modeling, the performance of easily interpretable models, such as logistic regression, is often comparable to that of complex extreme gradient boosting or neural network methods [115]. Third, complex models are susceptible to learning noise that may not generalize to a new dataset (*overfitting*) [116]. In this context, careful consideration of the available data and training plan (i.e., cross-validation), as well as strict separation of training and testing datasets, are warranted to maximize the external validity of new models.

### Geographic and temporal drift in model performance

The training of any clinical model should not stop at the time of deployment but rather continue by incorporating real-time data and updating its parameters to prevent a drift in performance when used in a different clinical, geographical, or temporal setting [117]. A notable example here is a widely implemented and proprietary EHR-embedded sepsis prediction model whose external performance was substantially different from the one reported by the model’s creators (AUROC of 0.63 from 0.76–0.83) when deployed across independent hospitals with heterogeneous patient populations [118].

### Promoting explainable AI

**Fig. 5:**
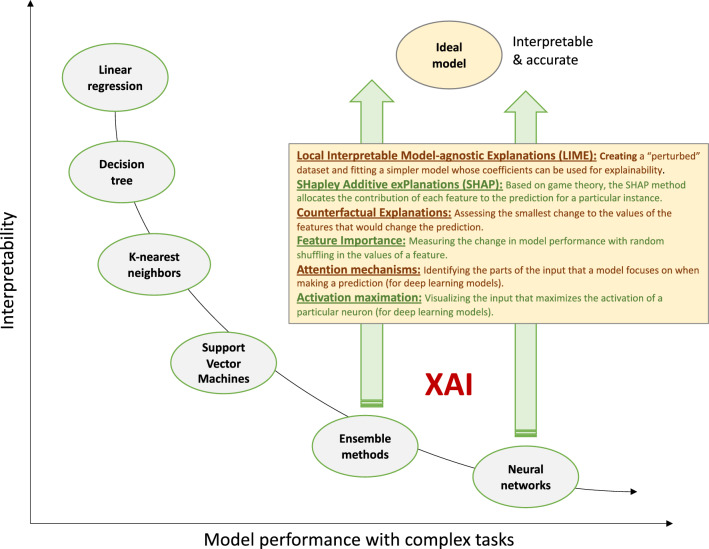
Explainability and interpretability of medical machine learning. Broadly speaking, more complex algorithms demonstrate better performance when dealing with complex tasks and data inputs. For instance, the recognition of cardiomyopathy using echocardiographic videos may require a deep learning algorithm to model the full extent of temporal and spatial features that carry diagnostic value, whereas predicting the risk of re-admission using electronic health record data may be modelled using generalized linear models. Simpler models, such as decision trees and linear models are intuitive and interpretable, whereas ensemble and neural network-based methods are too complex for the human mind to fully understand. Explainable artificial intelligence (XAI) methods aim to bridge this interpretability gap by offering direct or indirect insights into the inner workings of complex algorithms

To bridge the interpretability gap of complex “black box” algorithms, various approaches have emerged including Local Interpretable Model-agnostic Explanations (LIME) and SHapley Additive exPlanations (SHAP) [119, 120] (Fig. 5). Though such approaches are often imperfect, ensuring model explainability is not only a regulatory recommendation, but also enhances the adoption of the model in the real world. In a survey of 170 physicians, greater explainability of ML risk calculators was significantly associated with greater physician understanding and trust of the algorithm [121].

## Statistical, ethical and regulatory concerns: promoting equitable and safe AI use

### Ensuring good research practices

Clinical predictions rarely rely on a single factor and are most often multivariable by design. In 2015, to provide a standardized framework for the creation and reporting of such statistical models, the transparent reporting of a multivariable prediction model for individual prognosis or diagnosis (TRIPOD) statement was published [39]. This followed multiple reports, including systematic reviews of risk prediction models in type 2 diabetes, that showed widespread use of poor methods and reporting [122, 123] contributing to “avoidable waste” in research evidence [124]. Unfortunately, several subsequent reviews have shown poor adherence to these standards, particularly among studies using ML algorithms [125]. The increasing adoption of ML algorithms in clinical prediction modeling, despite the lack of clear incremental value beyond simpler methods such as logistic regression in most settings [115, 126], has prompted the original TRIPOD authors to update their statement (TRIPOD-AI, see [127]) researchers, clinicians, systematic reviewers, and policy-makers critically appraise ML-based studies. ML models should generally be used when processing large amounts of multi-dimensional or complex inputs (e.g. time-series from wearables, videos etc.), whereas head-to-head comparisons to traditional statistical models should be provided when feasible to assess the trade-off between performance, complexity, and interpretability.

### Mitigating bias through AI

Since ML models learn from existing data and care patterns, they can perpetuate human and structural biases [128, 129] (Table 1). Careful evaluation of the historical training data for health care disparities, ensuring that historically disadvantaged subgroups have adequate representation, review of model performance across key subgroups, and incorporating feedback from key stakeholders and patient representatives are some approaches that can be taken to mitigate bias [76, 128]. This is not a straightforward task and requires caution when assessing for confounders [130], since ML models have shown the ability to identify features such as race even when blinded to such labels [131].

**Table 1:** Types and examples of bias in medical artificial intelligence

Bias type	Definition: “A bias arising from…”	Example
Confirmation bias	A tendency to interpret data in a way that confirms our prior beliefs	A machine learning model confirms existing assumptions about certain broad phenotypic groups benefiting from a given therapy, potentially leading to unequal treatment and misdiagnosis
Sampling bias	Non-random sampling which limits the generalizability of an algorithm	Enrolling patients who visit a particular clinic or location may not represent the broader diabetes population
Algorithmic bias	The design and implementation of an algorithms that systematically discriminates against a given group	A blood pressure monitoring system that may provide consistently inaccurate readings for a given demographic group
Aggregation bias	Drawing misleading conclusions about individuals from group data	Concluding all patients with type 2 diabetes and hypertension benefit from a given medication without considering individual variations
Longitudinal data fallacy	Poor analysis of temporal data	Assessing quality of diabetes control and performing long-term risk prognostication using a single laboratory reading rather than long-term patterns
Implicit bias	Unintentional embedding of underlying biases and prejudices in algorithms	A model that is trained using records from a specific racial or ethnic group may make inaccurate predictions and disproportionately misclassify individuals from other racial groups as having higher or lower risk of diabetic complications contributing to healthcare disparities
User interaction bias	Both the user interface and the user's behavior	A diabetes management digital health app only collects voluntary input data, thus not capturing all relevant patient information
Presentation bias	How information is displayed to users	A patient may miss important information on an app due to the information's placement at the bottom of the screen
Emergent bias	Longitudinal changes in population, societal habits, norms, and practices over time	An outdated diabetes therapy might persist due to long-standing cultural beliefs
Evaluation bias	The process of model evaluation	The effectiveness of a novel antihyperglycemic therapy is evaluated against a benchmark that favors a particular demographic
Population bias	Differences in user characteristics between the training and the intended population	A diabetes management application initially tested among tech-savvy young adults may not adequately address the needs of older adults

### Navigating the regulatory framework

While clinical decision support (CDS) tools used to assist with electronic patient records, administrative tasks, medical device data, and models aimed at supporting a healthy lifestyle fall outside the FDA’s definition of a “device” [132], most diagnostic and predictive clinical models and CDS are regulated under the “*Software as Medicine Device (SaMD)*” umbrella and categorized as class I (low-risk), II (moderate-risk), III (high-risk) [20]. In the US, the FDA mandates that class III devices generally undergo a longer and more in-depth review process, known as pre-market authorization. However, for lower or intermediate-risk devices (class I and II), alternative pathways exist [20]. The 510(k) pathway requires manufacturers to show that the risk presented by their device is no greater than that of a substantially equivalent predicate device [20], whereas the de novo pathway is designed for class I or II devices without predicates [133]. While built to accelerate the regulatory process, the latter two pathways have been criticized on certain occasions for facilitating the clearance of devices based on faulty predicates [134]. The regulatory process is different in Europe, where lowest risk devices (class I) are the responsibility of the manufacturer, whereas class II and III devices are processed in a decentralized way through private “Notified Bodies”. Of the 124 AI/ML-based devices approved in the USA and Europe between 2015 and 2020, 80 were first approved in Europe [20]. The European Union’s General Data Protection Regulation (GDPR) further lists explainability as a requisite for any medical AI application [135]. Despite the above, there is no clear consensus as to whether regulatory bodies should require RCT-level evidence to support the effectiveness and safety of their proposed AI tools, even though recent studies have demonstrated the feasibility of testing the net clinical benefit of AI-based ECG and echocardiographic models in the context of pragmatic RCTs [136, 137].

## Conclusions

Rapid advances in AI and ML have revolutionized the field of medicine and have identified new ways to optimize the management of diabetes and its cardiovascular complications. Nevertheless, several challenges remain, ranging from standardizing the assessment of model performance along with model interpretability and explainability to mitigating bias during both development and deployment. Acknowledging these challenges and fostering a collaborative environment between clinicians, researchers, sponsors, and regulatory agencies is a prerequisite to harness the full potential of AI in catalyzing the transition towards a more patient-centered approach to the care of diabetes and CVD.

### Supplementary Information

**Additional file 1: Table S1.** Strengths and weaknesses of commonly used metrics in machine learning.

## Data Availability

Not applicable.

## Abbreviations

ADA: American Diabetes Association
AI: Artificial intelligence
AUROC: Area Under the Receiver Operating Characteristic Curve
CDS: Clinical decision support
CNN: Convolutional Neural Network
CVD: Cardiovascular disease
DL: Deep learning
ECG: Electrocardiography
EHR: Electronic health record
FDA: U.S. Food and Drug Administration
ML: Machine learning
NHANES: National Health and Nutrition Examination Survey
RCT: Randomized controlled trials
RNN: Recurrent neural network
XGBoost: EXtreme Gradient Boosting
