# Supplementary material for: Machine learning in precision diabetes care and cardiovascular risk prediction
Source: Cardiovasc Diabetol. 2023 Sep 25;22:259. doi: 10.1186/s12933-023-01985-3 (PMC10521578; doi:10.1186/s12933-023-01985-3)
Supplement: Supplementary file 1 — Additional file 1: Table S1. Strengths and weaknesses of commonly used metrics in machine learning. [file 12933_2023_1985_MOESM1_ESM.docx]

**Machine Learning in Precision Diabetes Care and Cardiovascular Risk Prediction**

**Additional file 1**

Evangelos K Oikonomou^1^, Rohan Khera^1,2,3,4*^

^1^Section of Cardiovascular Medicine, Department of Internal Medicine, Yale School of Medicine, New Haven, CT, USA

^2^Section of Health Informatics, Department of Biostatistics, Yale School of Public Health, New Haven, CT

^3^Section of Biomedical Informatics and Data Science, Yale School of Medicine, New Haven, CT

^4^Center for Outcomes Research and Evaluation, Yale-New Haven Hospital, New Haven, CT, USA

***Address for correspondence:**

Rohan Khera, MD, MS

195 Church St, 6th Floor, New Haven, CT 06510

203-764-5885; rohan.khera@yale.edu; @rohan_khera

**Table S1. Strengths and weaknesses of commonly used metrics in machine learning.**

| **Task** | **Metric** | **Brief definition** | **Strengths** | **Weaknesses** |
| --- | --- | --- | --- | --- |
| **CLASSIFICATION** | **Accuracy*** | $\frac{TP+TN}{TP+TN+FP+FN}$ | - Intuitive and easy to understand (the percentage of correct predictions). | - Misleading for imbalanced datasets.  - Does not incorporate the probability of the prediction (similar weights for high and low-confidence predictions). |
|  | **Sensitivity (Recall)*** | $\frac{TP}{TP+FN}$ | - Crucial where missing a positive case is costly. | - Can be high at the cost of many false positives.  - Does not account for true negatives. |
|  | **Specificity*** | $\frac{TN}{TN+FP}$ | - Crucial where false positives are costly. | - Does not provide insight on true positive rate.  - Can be high at the cost of many false negatives. |
|  | **Positive Predictive Value (PPV, Precision)*** | $\frac{TP}{TP+FP}$ | - Useful in contexts where false positives are costly.  - Depends on the prevalence of the label of interest. | - Can be misleading in highly imbalanced datasets  - Does not account for false negatives. |
|  | **Negative Predictive Value (NPV)*** | $\frac{TN}{TN+FN}$ | - Useful in contexts where false negatives are costly.  - Depends on the prevalence of the label of interest. | - Can be misleading in highly imbalanced datasets.  - Does not account for false positives. |
|  | **AUROC** | *The area under a curve that tracks the true versus false positive rate across different thresholds.* | - It is threshold-invariant (measures the quality of predictions independent of any specific threshold) | - May be over-optimistic for imbalanced datasets.  - It is scale invariant (measures how well predictions are ranked, not their calibration and absolute values) |
|  | **AUPRC** | *The area under a curve that tracks the precision (PPV) versus recall (sensitivity).* | - Focuses on the performance of the positive class.  - It is threshold-invariant (measures the quality of predictions independent of any specific threshold) | - Not as interpretable as AUROC.  - By focusing on the performance of the positive class, it tends to ignore the negative class. |
|  | **F1 Score*** | $2\times\frac{Precision\times Recall}{Precision+Recall}$ | - Useful for imbalanced datasets, where recall and precision must be optimized simultaneously (represents the harmonic mean of the two) | - Ignores the true negatives; misleading in unbalanced datasets.  - Lack of symmetry (value changes if positive and negative labels are flipped). |
| **REGRESSION** | **MSE (mean squared error)** | $\frac{1}{n}\sum_{i=1}^{n} \left( y_{i}-\hat{y_{i}} \right)^{2}$ | - Punishes large errors more due to squaring.  - Differentiable, making it suitable for optimization. | - Sensitive to outliers.  - Scale-dependent (not always intuitive). |
|  | **MAE (mean absolute error)** | $\frac{1}{n}\sum_{i=1}^{n} \left\vert y_{i}-\hat{y_{i}} \right\vert$ | - Linear penalty for errors.  - Less sensitive to outliers compared to MSE. | - Might be less stable for optimization than MSE. |
|  | **R^2^** | $1-\frac{SS_{\text{residuals}}}{SS_{\text{total}}}$ | - Provides proportion of variance explained by the model.  - Value between 0 (bad) and 1 (perfect). | - Can be misleading (a low R^2^ doesn’t necessarily mean bad fit).  - Not always indicative of prediction errors. |
| *threshold-dependent metrics; FN/FP: false negatives/positives; n = total number of observations; $SS_{\text{residuals}}$ = the sum of the squared differences between the actual values and the predicted values (residuals); $S_{\text{total}}$ = sum of the squared differences between the actual values and the mean of the actual values; TN/P: true negatives/positives; $y_{i}$ = actual value; $\hat{y_{i}}$ = predicted value. | | | | |
